# Healthcare trainees’ Hepatitis B surface antibodies in the times of universal vaccination: a cross-sectional study

**DOI:** 10.1017/ash.2025.10146

**Published:** 2025-10-06

**Authors:** Cynthia Isabel Ortiz-Lopez, Adrian Camacho-Ortiz, Magaly Padilla-Orozco, Paola Bocanegra-Ibarias, Mariana Contreras-Ruiz, Mariana Ramirez-Yañez, Eduardo Perez-Alba

**Affiliations:** 1 Servicio de Infectología, Hospital Universitario “Dr Jose Eleuterio Gonzalez”, Universidad Autonoma de Nuevo Leon, Monterrey, Mexico; 2 Coordinacion de Epidemiologia Hospitalaria, Hospital Universitario “Dr Jose Eleuterio Gonzalez”, Universidad Autonoma de Nuevo Leon, Monterrey, Mexico

## Abstract

With widespread childhood vaccination, young healthcare workers likely have HBV immunity. This cross-sectional, serological aimed to determine the proportion of healthcare trainees with anti-HBs ≥10 mIU/mL after accidental exposure and compare it to self-reported vaccination. We found 77.2% prevalence of anti-HBs, thus, awareness of immunity is vital in resource-limited settings.

## Introduction

Hepatitis B virus (HBV) is a leading cause of liver disease and a global health that can lead to cirrhosis and cancer. An estimated 1.2 million infections occur annually,^1^ thus, in the context of a high efficacy HBV vaccine the World Health Organization (WHO) issued recommendations in 1997 for it to be included in all immunization programs.^2^

HBV can be transmitted in healthcare settings through contact with body fluids, such as blood or saliva, via contaminated needles, syringes, or sharp objects. Given the morbidity associated with occupational exposure to HBV in healthcare workers without immunity, contact with body fluids is considered a serious concern.^3^ However, with the widespread implementation of childhood HBV vaccination, it’s possible that most of the future healthcare personnel could have protective antibodies.

Both the United States’ Center for Disease Control and the WHO recommend a complete HBV vaccination schedule followed by antibodies against Hepatitis B surface antibodies (anti-HBs) measurement.^3,4^ Several studies have examined student’s vaccination status; however, few have explored whether these individuals already possess immunity from childhood vaccinations.^5–7^

Resource-limited countries may have limited strategies to either re-vaccinate their healthcare personnel and/or measure anti-HBs. Therefore, the aim of this study was to assess anti-HBs levels in healthcare trainees whose age suggests they should be protected by childhood vaccination (Mexico implemented universal HBV vaccination in 1999 and achieved over 95% coverage by 2006, meaning these adults would have received the vaccine during childhood).

## Methods

Authorization by our Hospital’s Ethics Committee was obtained to conduct a cross-sectional, descriptive serological survey among healthcare trainees enrolled at Hospital Universitario “Dr. José Eleuterio González” in Mexico, who reported an occupational exposure. Inclusion criteria comprised: being enrolled as a healthcare trainee aged 18 to 25 years at the university hospital who suffered an occupational exposure (medical student, medical resident, nursing student, respiratory technician, dentistry student). Additionally, the trainee must be willing to provide informed consent, will to disclose self-reported vaccination status, and acceptance of blood sample collection. A complete HBV vaccination schedule was defined as having received three or more HBV vaccine doses, incomplete as receiving two or fewer doses, and unknown if the participant couldn’t recall their vaccination history.

Recruitment took place between August 2023 and October 2024. Blood samples were drawn within twenty-four hours after the exposure, and centrifuged at 3,000 rpm for ten minutes. Anti-HBs levels were measured using chemiluminescence immunoassay on the ARCHITECT System (Abbott Diagnostics®, Illinois, USA) according to manufacturer’s instructions. This assay has a specificity of 99.67% and a sensitivity of 97.5%, with a detection range of 2.50–1,000.00 IU/L.^8^ A level below 10 mIU/mL was considered negative; titers ≥10 mIU/mL and <100 mIU/mL were low (but protective), ≥100 mIU/mL, and <1,000 mIU/mL were moderate, and ≥ 1,000 mIU/mL was a hyper-response.

Descriptive statistics were used to calculate medians, interquartile ranges (IQR), frequencies, and percentages. χ^2^ test was applied to compare gender, vaccination status, type of occupational exposure, and type of body fluid exposure. Student’s t-test was used to compare age and anti-HBs titers. A *P* value ≤.05 was considered statistically significant. Data were collected using Microsoft Excel and analyzed with SPSS Statistics version 30..0.0 (IBM, New York, USA).

## Results

A total of 486 healthcare students were screened and 414 didn’t meet inclusion criteria. We enrolled 72 students, however, six participants didn’t attend the blood drawn (Supplementary Figure 1). Thus, 66 participants were included: 20 (30.3%) with a complete vaccination status, 25 (37.8%) with an incomplete status, and 21 (31.8%) with an unknown status. Most were women (44, 66.6%) and nursing students (31, 46.9%); the median age was 22 years (IQR 4). The most common kind of exposure, type of fluid, and sharp were needle puncture (78.7%), blood (92.3%), and exposure to syringes or needles (78.9%), respectively. The most frequent location for these exposures were patient rooms (40.9%) (Supplementary Table 1).

Overall, most students had anti-HBs titers ≥10 mIU/mL (*n* = 51; 77.2%). Trainees who had detectable anti-HBs were predominantly women (*n* = 36, 81.2%) and nursing students (*n* = 26, 83.8%). Those immune were distributed similarly within the different vaccination status groups: complete (*n* = 16, 80%), incomplete (*n* = 20, 80%), and unknown (*n* = 15, 71.4%) (Table 1).

**Table 1.** Comparison of characteristics of healthcare students with antibodies against hepatitis B virus surface antigen

Characteristics	Total *n* = 66 (%)	Protective Anti-HBs *n* = 51 (%)	Negative Anti-HBs *n* = 15 (%)	*P*
Sex Female (%) Male (%)	44 (66.6) 22 (33.3)	36 (81.8) 15 (68)	8 (18.2) 7 (31.8)	.21
Healthcare degree Medicine (%) Nursing (%) Other (%)	29 (43.9) 31 (46.9) 6 (9)	21 (72.9) 26 (83.8) 4 (66.6)	8 (27.5) 5 (16.1) 2 (33.3)	.37
HBV vaccination status Complete (%) Incomplete (%) Unknown (%)	20 (30.3) 25 (37.8) 21 (31.8)	16 (80) 20 (80) 15 (71.4)	4 (20) 5 (20) 6 (28.5)	.74
Type of occupational exposure Puncture (%) Splash (%) Other (%)	52 (78.7) 13 (19.6) 1 (1.5)	42 (80.7) 9 (69.2) 0	10 (19.2) 4 (30.7) 1 (100)	.12

*Note*: a: chemist, laboratory technician, criminology, dentistry. Anti-HBs: Hepatitis B surface antibody; protective: ≥10 mIU/ml; negative: <10 mIU/ml. Chi^2^ was used for sex, healthcare degree, HBV vaccination status and type of occupational accident. A *P* value < .05 was statistically significant.

Amid those who reported incomplete vaccination and had protective anti-HBs, most were women (*n* = 17, 77.2%, *P* = .04). In men, no significant differences were observed in the presence of protective titers, regardless of vaccination status. HBV immunity between nursing and medical students wasn’t different, regardless of their vaccination status (Supplementary Table 2).

There was no significant difference in protective anti-HBs titers (low, moderate, or hyper-response) relative to the number of self-reported vaccine doses. Nearly all participants with immunity had anti-HBs levels >100 mIU/mL, and a third of them (*n* = 17/51) had a hyper-response that was not related to either vaccination status (Supplementary Table 3).

## Discussion

Childhood vaccination has been proven effective even in countries with a high prevalence of HBV infection.^9^ In our study, we screened healthcare trainees born after the introduction of universal vaccination to assess their acquired immune status. This knowledge aids in resource allocation when planning revaccination strategies, especially in public healthcare policies of resource-limited countries.

While most of our participants reported incomplete vaccination, the seroprevalence of anti-HBs was 77.2%, higher than the 47% reported in a previous study of healthcare students in our country.^6^ Lack of universal protection could be attributed to different reasons: (1) nonresponse to the vaccine, (2) different brands of vaccines with different immunogenicity used through time, (3) inaccurate reports of universal vaccination coverage.

Among our population with an incomplete vaccination history, 80% had protective anti-HBs titers, similar to what has been reported in Brazil (71.4–77%).^10,11^ Furthermore, 71.4% of our participants with unknown vaccination status also had protective anti-HBs titers. It is noteworthy that four trainees with self-reported complete vaccination (23.8%) had titers <10 mIU/mL, which may indicate nonresponse to vaccination or inaccurate recall of vaccination history. As in many other countries, Mexico has yet to implement a national electronic vaccine registry which could reliably confirm a person’s history.

Lack of complete immunization remains a global issue. Success rates for completing a three-dose vaccination schedule have varied from 5.5% to 16.7% in countries with similar HBV prevalence.^10,12,13^ We documented an unacceptably high percentage of students (37.8%) with an incomplete vaccination schedule and 31.8% had unreliable vaccination recall. While a study by Trevissan *et al*. on medical students found that 91.8% had detectable anti-HBs with a positive predictive value of 93.2% for self-reported vaccination status,^14^ we highlight the importance of a potential recall bias and that self-reported vaccination status is unreliable. We also acknowledge two limitations in our study: the lack of medical and vaccination records, and the use of different HBV vaccine brands nationwide over the past years, which may have contributed to immunological heterogeneity.

Although the percentage of healthcare students with protective anti-HBs has increased, public health vaccination coverage remains suboptimal. It shouldn’t be assumed that high vaccination coverage guarantees protection against HBV in healthcare workers. While this may change in the future, measuring anti-HBs in at-risk populations should be mandatory.

In conclusion, healthcare trainees born between 1999 and 2005 had approximately 70% anti-HBs titers ≥10 mIU/mL, regardless of their reported vaccination history. However, about one in four had titers below the protective threshold, despite self-reports of complete vaccination schedule.

Preliminary data from this work has been presented as a poster at the “XXVIII Congreso Internacional de AMEIN” in March 2024.

## Supporting information

10.1017/ash.2025.10146.sm001 Ortiz-Lopez et al. supplementary material 1 Ortiz-Lopez et al. supplementary material

10.1017/ash.2025.10146.sm002 Ortiz-Lopez et al. supplementary material 2 Ortiz-Lopez et al. supplementary material

10.1017/ash.2025.10146.sm003 Ortiz-Lopez et al. supplementary material 3 Ortiz-Lopez et al. supplementary material

10.1017/ash.2025.10146.sm004 Ortiz-Lopez et al. supplementary material 4 Ortiz-Lopez et al. supplementary material

10.1017/ash.2025.10146.sm005 Ortiz-Lopez et al. supplementary material 5 Ortiz-Lopez et al. supplementary material

## Data Availability

The database used and analyzed in this study is available from the corresponding author upon reasonable request.

## Supplementary material

The supplementary material for this article can be found at https://doi.org/10.1017/ash.2025.10146.
