# Supplementary material for: Healthcare trainees’ Hepatitis B surface antibodies in the times of universal vaccination: a cross-sectional study
Source: Antimicrob Steward Healthc Epidemiol. 2025 Oct 6;5(1):e247. doi: 10.1017/ash.2025.10146 (PMC12509149; doi:10.1017/ash.2025.10146)
Supplement: Ortiz-Lopez et al. supplementary material 2 — Ortiz-Lopez et al. supplementary material [file S2732494X25101460sup002.docx]

Supplementary Figure 1. Flow chart of participation of healthcare personnel in training who reported occupational exposure.
