# Supplementary material for: Healthcare trainees’ Hepatitis B surface antibodies in the times of universal vaccination: a cross-sectional study
Source: Antimicrob Steward Healthc Epidemiol. 2025 Oct 6;5(1):e247. doi: 10.1017/ash.2025.10146 (PMC12509149; doi:10.1017/ash.2025.10146)
Supplement: Ortiz-Lopez et al. supplementary material 3 — Ortiz-Lopez et al. supplementary material [file S2732494X25101460sup003.docx]

Supplementary Table 1. Characteristics of healthcare trainees and their occupational exposures.

| Characteristics  n=66 | Frequency (%) |
| --- | --- |
| Sex  Female | 44 (66.6) |
| Age [median (IQR)] years | 22 (4) |
| Healthcare degree  Medicine  Nursing  Other | 29 (43.9)  31 (46.9)  6 (9) |
| HBV vaccination status  Complete  Incomplete  Unknown | 20 (30.3)  25 (37.8)  21 (31.8) |
| Type of occupational exposure  Puncture  Splash  Other^a^ | 52 (78.7)  13 (19.6)  1 (1.5) |
| Type of sharps  Needle, disposable syringe  Suture needle  Other^b^ | 41 (78.8)  9 (17.3)  2 (3.8) |
| Type of bodily fluid  Blood or blood products  Saliva | 12 (92.3) 1 (7.6) |
| Location of exposure  Patient room Emergency department Operation room  Laboratory  Consult office  Other^c^ | 27 (40.9) 19 (28.7)  10 (15.5)  3 (4.5)  2 (3)  5 (7.5) |
| *Note*:^a^ Bite; ^b^ Bone fragment, glass test tube; ^c^ Dentistry, Mortuary | |
