# Supplementary material for: Healthcare trainees’ Hepatitis B surface antibodies in the times of universal vaccination: a cross-sectional study
Source: Antimicrob Steward Healthc Epidemiol. 2025 Oct 6;5(1):e247. doi: 10.1017/ash.2025.10146 (PMC12509149; doi:10.1017/ash.2025.10146)
Supplement: Ortiz-Lopez et al. supplementary material 4 — Ortiz-Lopez et al. supplementary material [file S2732494X25101460sup004.docx]

Supplementary Table 2. Comparison between gender, career and HBV vaccination status with production of anti-HBs.

| Characteristics | Total  N=66 (%) | Protective Anti-HBs  n=51 (%) | Negative Anti-HBs  n=15 (%) | *P* |
| --- | --- | --- | --- | --- |
| Female  Complete vaccination status (%)  Incomplete vaccination status (%)  Unknown vaccination status (%) | 44 (66.6)  8 (18.1)  22 (50)  14 (31.8) | 36 (81.8)  6 (75)  17 (77.2)  13 (92.8) | 8 (18.2)  2 (25)  5 (22.7)  1 (7.1) | .30 .04  .24 |
| Male  Complete vaccination status (%)  Incomplete vaccination status (%)  Unknown vaccination status (%) | 22 (33.3)  12 (54.5)  3 (13.6)  7 (31.8) | 15 (68)  8 (66.6)  3 (100)  2 (28.5) | 7 (31.8)  2 (16.6)  0  5 (71.4) | .13 .66 .45 |
| Medicine  Complete vaccination status (%)  Incomplete vaccination status (%)  Unknown vaccination status (%) | 29 (43.9)  13 (44.8)  6 (20.6)  10 (34.4) | 21 (72.9)  9 (69.2)  5 (83.3)  7 (70) | 8 (27.5)  4 (30.7)  1 (16.6)  3 (30) | .50 .56 .97 |
| Nursing  Complete vaccination status (%)  Incomplete vaccination status (%)  Unknown vaccination status (%) | 31 (46.9)  5 (16.1)  16 (51.6)  10 (32.2) | 26 (83.8)  5 (100)  13 (81.2)  8 (80) | 5 (16.1)  0  3 (18.7)  2 (20) | .88 .92 .81 |
| Note: Anti-HBs: Hepatitis B surface antibody; protective: ≥10 mIU/ml; negative: <10 mIU/ml. Chi2 was used for gender, healthcare degree, HBV vaccination status and detection of Anti-HBs. A *P* value <.05 was statistically significant. | | | | |
