# Supplementary material for: Healthcare trainees’ Hepatitis B surface antibodies in the times of universal vaccination: a cross-sectional study
Source: Antimicrob Steward Healthc Epidemiol. 2025 Oct 6;5(1):e247. doi: 10.1017/ash.2025.10146 (PMC12509149; doi:10.1017/ash.2025.10146)
Supplement: Ortiz-Lopez et al. supplementary material 5 — Ortiz-Lopez et al. supplementary material [file S2732494X25101460sup005.docx]

Supplementary Table 3. Comparison between levels of anti-HBS titers and HBV vaccination status.

| HBV vaccination status | Total Protective  Anti-HBs (%) | low  (%) | *P* | Moderate  (%) | *P* | Hyper-response  (%) | *P* |
| --- | --- | --- | --- | --- | --- | --- | --- |
| Complete  Incomplete  Unknown | 16 (80)  20 (80)  15 (71.4) | 4 (25)  3 (15)  6 (40) | .96  .22  .21 | 6 (37.5)  10 (50)  5 (33.3) | .83  .26  .34 | 6 (37.5)  7 (35)  4 (26.6) | .60  .74  .39 |
| *Note*: HBV: Hepatitis B virus, Anti-HBs: hepatitis b surface antibody. Protective: ≥10 mIU/ml: low ≥10 mIU/ml and < 100 mIU/ml; moderate: ≥100 mIU/ml and < 1000 mIU/ml; hyper-response: ≥ 1000 mIU/ml. Chi^2^ was used for HBV vaccination status and titers of anti-HBs. A *P* value <.05 was statistically significant. | | | | | | | |
